# A data-driven approach to quantify disparities in power outages

**DOI:** 10.1038/s41598-023-34186-9

**Published:** 2023-05-04

**Authors:** Arkaprabha Bhattacharyya, Makarand Hastak

**Affiliations:** 1 Lyles School of Civil Engineering, Purdue University, 550 Stadium Mall Dr., West Lafayette, IN 47907 USA; 2 Division of Construction Engineering and Management, Civil Engineering, Purdue University, 550 Stadium Mall Dr., West Lafayette, IN 47907 USA

**Keywords:** Energy and society, Socioeconomic scenarios, Sustainability

## Abstract

This research proposes a data-driven approach to identify possible disparities in a utility’s outage management practices. The approach has been illustrated for an Investor-Owned Utility located in the Midwest region in the U.S. Power outage data for approximately 5 years between March 2017 and January 2022 was collected for 36 ZIP/postal codes located within the utility’s service territory. The collected data was used to calculate the total number of outages, customers affected, and the duration of outages during those 5 years for each ZIP code. Next, each variable was normalized with respect to the population density of the ZIP code. After normalizing, a K-means clustering algorithm was implemented that created five clusters out of those 36 ZIP codes. The difference in the outage parameters was found to be statistically significant. This indicated differential experience with power outages in different ZIP codes. Next, three Generalized Linear Models were developed to test if the presence of critical facilities such as hospitals, 911 centers, and fire stations, as socioeconomic and demographic characteristics of the ZIP codes, can explain their differential experience with the power outage. It was found that the annual duration of outages is lower in the ZIP codes where critical facilities are located. On the other hand, ZIP codes with lower median household income have experienced more power outages, i.e., higher outage counts in those 5 years. Lastly, the ZIP codes with a higher percentage of the White population have experienced more severe outages that have affected more customers.

## Introduction

Power outages happen every year, and millions of people suffer from them^1^. Power outage restoration is a very complex, multi-objective, multi-stage, multi-variable, and multi-constraint optimization issue and is full of non-linearity and uncertainty^2^. During any large-scale power outage, utilities prioritize territories based on the presence of different critical facilities^3^. Critical facilities such as hospitals, 911 centers, fire stations, etc., often appear at the top of utilities’ restoration priority list. Once those are restored, they focus on the downed lines. After those drowned lines are restored, utilities shift their focus toward the households.

There is a plethora of evidence that points towards disparities in the power outage restoration process after major disasters^3–7^. These restorations are often biased against minority and low-income communities. Not only after major disasters, but utilities should also ensure equitable power outage management practices during normal operations^7^. While duration has been a preferred choice in analyzing the disparity in post-disaster power outages, other outage parameters, such as the number of outages in a year, the number of customers affected, etc., should also be considered for analyzing disparities during normal operations. These parameters collectively represent the extent of power outages faced by different communities^8,9^.

As explained earlier, utilities’ restoration practices often prioritize critical facilities over households, which can cause differences in outage duration between areas with and without critical facilities. However, the extent of a power outage can be dissimilar in different areas due to reasons other than the presence of critical facilities and the above-explained disparities. For instance, factors such as population density, location of power lines (e.g., underground vs. overhead), exposure to natural hazards, etc., can influence the extent of power outages in an area^8,9^. When these factors are controlled, there should not be much difference in the extent of power outage between different areas within a utility’s service territory. If that difference is statistically significant, it reflects differential experience with a power outage in different areas. It can be further argued that the spatial difference in the extent of the power outage is due to the difference in the vulnerability of the grid components in different locations. But the spatial difference in the vulnerability of grid components among areas within a utility’s service territory with similar population density, exposure to natural hazards, etc., also indicates disparities in infrastructure management.

Regardless of people’s background, education, or ethnicity, everyone should have equitable access to infrastructures and services such as electricity. The absence of this equitable service is defined as disparity in the context of this research. This disparity can be either intentional or unintentional. They might be due to implicit bias^10^. Whatever the reasons are, utilities should periodically assess the performance of their outage management practice to identify any disparity. On the other hand, the regulatory commissions should also oversee that there is no bias in a utility’s outage management practice during normal operations. Most of the previous research has analyzed the disparities in post-disaster outage management practices. Disparities in the outage management practices during normal operations are relatively underexplored, which indicates the need for this research.

This research fulfills that gap by proposing a data-driven approach that utilizes a utility’s historical data to identify the power outage disparity in its service territory. The proposed approach has been demonstrated for an investor-owned utility (IOU) located in the Midwest region of the U.S. Power outage data for 5 years between 2017 and 2021 was collected, and the data was aggregated at ZIP or postal code level. First, a K-means clustering algorithm was utilized. The algorithm created different clusters of ZIP codes with similar outage characteristics. Next, the inter-cluster difference of three popular reliability indices: System Average Interruption Duration Index (SAIDI), System Average Interruption Frequency Index (SAIFI), and Customer Average Interruption Duration Index (CAIDI), were tested using the Kruskal–Wallis test. Lastly, three Generalized Linear Models (GLM) were developed for the annual power outage count, total customers affected annually, and the total annual outage duration for each ZIP code and normalized with respect to population density to explore the influence of the socioeconomic and demographic factors, and presence of critical facilities such as hospitals, 911 centers, and fire stations on them.

## Research background

Ensuring equal access to clean water and sanitation, affordable clean energy, and reducing inequalities are three of the seventeen Sustainable Development Goals (SDGs) set by the United Nations^11^. Building on the SDGs, discussions on the inequality in access to urban infrastructures have gained popularity in recent times. The disparity in the accessibility to urban infrastructures contributes to growing inequalities among communities^12^. Nicoletti et al.^12^ have found that communities that have a larger share of minorities earn less and have a lower number of individuals with a university degree have low access to urban infrastructures. Braun et al.^13^ analyzed access to bike lanes in 22 U.S. cities and found that communities with lower socioeconomic status have lesser access to bike lanes. Inadequate access to water and sanitation is prevalent among communities in the U.S. with higher American Indian and Alaskan Native households^14^. Similar evidence was also presented in^15,16^.

Like water and sanitation, energy is one of the most essential infrastructures. The disparity in the energy insecurity, i.e., the uncertainty that a household can pay its energy bills^17^ is extensively discussed in the existing literature. Graff and Carley^17^ have found substantial difference in the percentage income that is spent on energy bills among different socioeconomic and demographic households in urban and rural U.S. On average, low-income households living in the rural areas spent 9% of their income on energy bills^17^. Memmott et al.^18^ have found that energy insecurity is highly prevalent in the low-income households in the U.S. that are at or below 200% of the federal poverty line. They also found that Black and Hispanic households are more likely to face service disconnection and energy insecurity. Similar evidence was found after hurricane Sandy in 2012^19^. Baker et al.^20^ have claimed that this issue of energy insecurity among low-income households of color has exacerbated during the COVID-19 pandemic. Steele and Bergstrom^21^ have developed an index that can measure the extent of energy insecurity among the U.S. households. Their research showed that in 2015, between 9% and 22% of the households they surveyed were energy insecure. Other studies investigating energy insecurity among low-income households can be found in^22–26^.

Frequent and large-scale power outages can have devastating economic and social impacts on a society. Bhattacharyya et al.^1^ showed that an annual production loss of 1% in the U.S. utility sector due to severe weather events can cause the U.S. Gross Domestic Product billions of dollars of loss. On the other hand, power outages can have severe impacts on residents’ lives, commercial activities, public services, and many more^27–29^. Disparities in the power outage restoration has also been discussed in the existing literature. In the aftermath of major disasters, it is often reported that existing power restoration processes place larger burden on the marginalized communities. Tormos-Aponte et al.^3^ have analyzed power outage data in Puerto Rico after hurricane Maria in 2017. They found that the communities with ties to the ruling party in Puerto Rico are more likely to be prioritized during disaster relief. Ulak et al.^30^ have analyzed power outage data in Tallahassee, Florida after hurricane Hermine in 2016. They have found that the extent of power outage represented as percentage of customers affected in a census block was higher in the areas with lower median family income. During the severe winter storm “Uri” in Texas in February 2021, similar disparities were reported. Flores et al.^31^ have analyzed hourly county level power outage data and found that counties with more Hispanic residents tended to experience more severe power outage. They did not find socioeconomic and medical disparities in the power outage. Another research has found similar evidence from that winter storm. Nejat et al.^32^ have also analyzed county level outage data and found a negative correlation between the ratio of outage and counties’ population of non-Hispanic whites and median household income. Similar evidence was also presented in^5^. They have also found significant disparity in the extent and duration of power outage experienced by low-income and minority groups. Although similar disparities were reported for other types of natural hazards such as hurricanes, severe storms, etc., in^3,33,34^, the disparity during the winter storm “Uri” is particularly insightful and at the same time problematic. This is because those rolling outages were planned to prevent the grid from collapsing^29,32,35^.

Despite their merits, these studies that have found evidence of bias against low-income population of color are event specific^31–34^. Utilities provide service 24-7. Therefore, the performance of their outage management practice should also be judged during normal operations^7^. Moreover, all the existing evidence are for the outages due to weather related events that normally cause prolonged power outages^1^. But there are other types of outages caused by equipment failures, intentional attacks, accidents, etc. Those outages should also be considered in judging the performance of the utility’s outage management practice. Although outage duration is a preferred choice for analyzing disparities in power outage, other dimensions such as outage count, the number of customers affected is relatively underexplored. To the best of the authors’ knowledge, no previous research has analyzed the disparity in power outage management practice with these three dimensions, i.e., outage count, number of customers affected, and duration of the outage collectively while considering a longer time frame so that outages of different types and causes are not excluded. This research is aimed to bridge that gap.

## Research data

The power outage data was collected for an Investor-Owned Utility located in the Midwest Region of the U.S. The utility serves around 150,000 customers in the region. The power outage data for 36 ZIP codes within its service territory was collected for approximately 5 years between March 2017 and January 2022. The data was collected from the utility’s Twitter account. The utility tweets about the power outages that occur within its service territory. The tweets contain multiple information on the outages such as the address, number of customers affected, possible causes, etc. It should be noted that the tweets’ structures were not regular and not all tweets contained all these information. Additionally, the utility sometimes retweets to inform the progress and/or completion of the restoration process. The difference between the original tweet notifying the outage and the retweet notifying completion of restoration is used as the outage duration in this research. Again, it does not always retweet when the outages are restored.

This research utilized Twitter’s Application Programming Interface (API) to download tweets between March 2017 and January 2022 from the Utility’s Twitter account. It should be noted that Twitter only allows downloading a limited number of tweets from a particular account. The collected tweets were utilized to extract the power outage data. Tweets that did not mention the number of customers affected due to the outage were discarded. After discarding those, 594 tweets were left. Each of these tweets represented one power outage. The addresses mentioned on the tweets were used in Google Maps to find the ZIP codes of the outage locations. There were fifty tweets that did not mention the outage location. For those cases, it was assumed that the outage was spread across the service territory. So, the number of customers affected was evenly distributed among the 36 ZIP codes within the utility’s service territory.

Among the 594 outages, 275 had the duration information, i.e., the utility retweeted about the completion of restoration. Among those 275 outages, 40 did not have the affected customer information. For those 40 cases, the missing data was replaced by the average customers affected in that ZIP code during that year. Lastly, among those 275 outages, 15 did not have the location information. For those, it was assumed that the outage affected all the ZIP codes in the utility’s service territory. The causes of power outage were only available for 182 cases. The causes included severe weather events, falling trees, wildlife, equipment failures, etc. Majority of the outages were associated with equipment failures (30%), which was followed by severe weather events (20%), vegetation related events (18%), accident (9%), etc.

**Figure 1:**
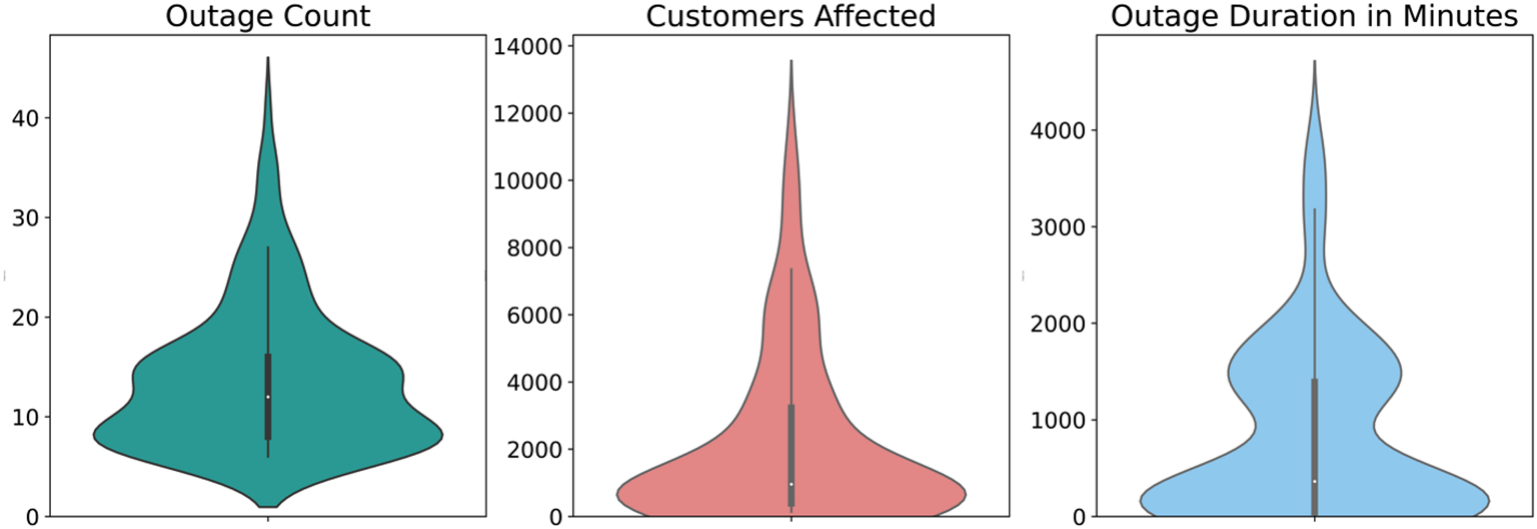
Distributions of the power outage parameters among the 36 ZIP Codes.

Next a panel data, which is also known as longitudinal data or cross-sectional time series data, was created by aggregating the three parameters of power outages, i.e., the outage count, the number of customers affected, and the outage duration in minutes for each ZIP code for each year between 2017 and 2021. So, the data had the three outage parameters for 36 ZIP codes for 5 years, i.e., 180 datapoints. Figure 1 shows the density plots. It can be seen that all three variables have long tails, i.e., they are right skewed. The skewness coefficients for outage count, customers affected, and the outage duration were 1.26, 1.63, and 1.23, respectively. The median values of the outage count, number of customers affected, and the outage duration were 12, 959, and 364 min, respectively.

## Research methodology

**Figure 2:**
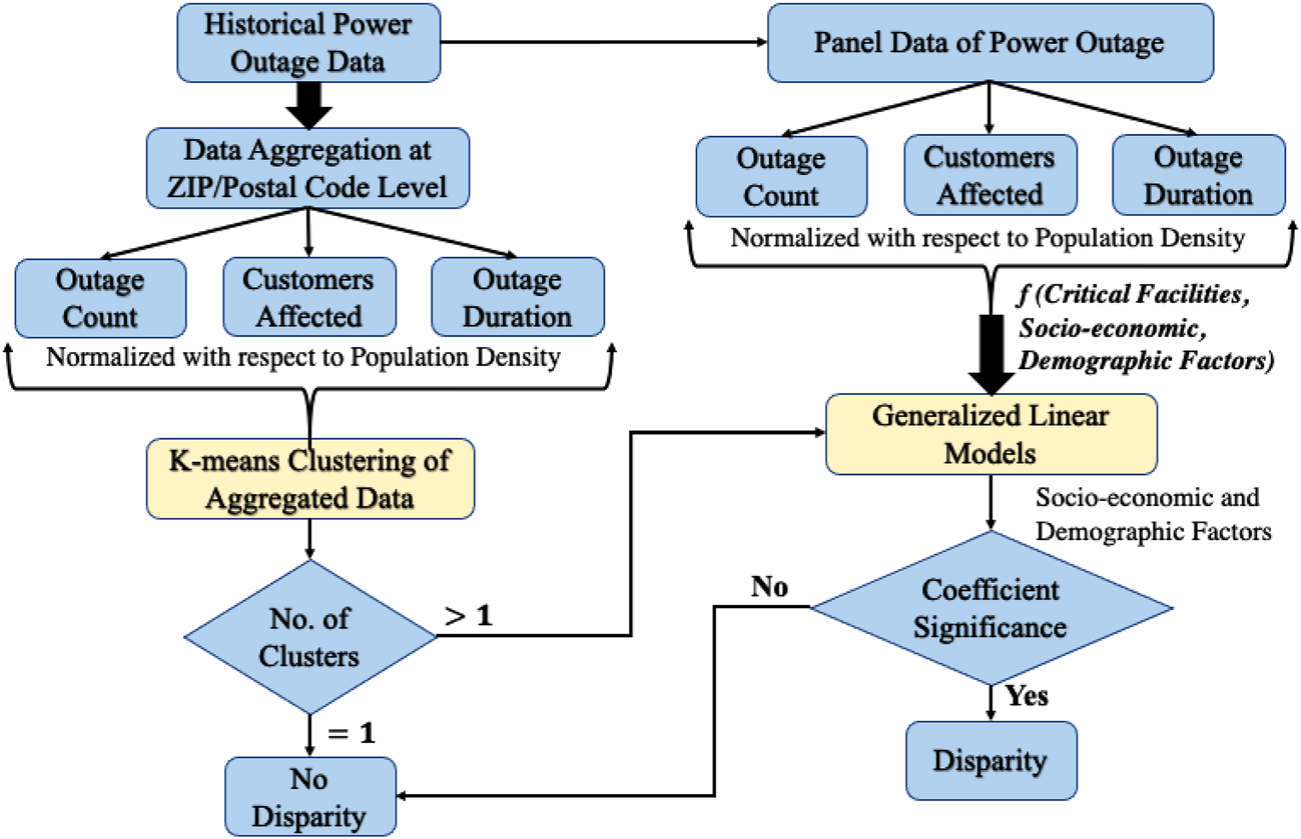
Power outage disparity quantifying framework.

There are two objectives of this research. (1) to propose a data driven approach that can be used to quantify disparities in the power outage management practice of a utility and (2) to investigate if the socioeconomic, and demographic characteristics can explain the difference in the extent of power outages between different areas within the utility’s service territory. The proposed approach of quantifying disparities in power outage and recovery is shown in Fig. 2 and based on the following assumption. If the determinants of the differentials of power outage such as population density, location of power lines (underground or overhead), exposure to natural hazards, etc., can be controlled, the difference in the outage parameters among different areas within the utility’s service territory should not be statistically significant. If the difference is statistically significant, it indicates differential experience with power outages in different areas within the service territory. It should be clarified that the proposed approach does not investigate or infer the causes behind such disparities. It rather facilitates the process to identify them.

The 36 ZIP codes within the utility’s service territory covers approximately 1912 square miles of area, which is only 5.3% of the state’s area where it is located. Therefore, it is safe to assume that there is not much difference in the exposure to natural hazards. Moreover, it has been explained before that severe weather events contributed 20% of the power outages. Again, population density is another important factor that influences the extent of power outage experienced by different locations^8^. Population density also influences the location of distribution lines. Densely packed urban areas tend to have more underground electric lines^8,29^. Mukherjee et al.^8^ stated that cities located in the Midwest region in the U.S. such as Indianapolis, Evansville, etc., have underground power lines in the downtown areas where the population density is normally higher. Thus, population density also serves as a proxy variable for the location of the power lines. To control the effects of population density and location of distribution lines, the outage parameters, i.e., outage count, number of customers affected, and the outage duration were normalized with respect to the population density of the ZIP code. Next, the normalized outage parameters were aggregated for 5 years, i.e., for each ZIP code the sum of values of the normalized outage parameters for 5 years was calculated from the panel data. It was used to conduct the clustering analysis. Spatial analysis at the ZIP code level is common in the literature and can be found in^36–41^. Table 1 summarizes the descriptive statistics of the normalized outage parameters.

**Table 1:** Descriptive statistics of the normalized outage parameters.

	No. of outages	No. of customers affected	Duration in minutes
No. of datapoints	36	36	36
Mean	1.03	93.56	63.07
Standard Deviation	1.14	95.01	66.46
Minimum	0.03	6.32	1.60
Maximum	5.93	441.75	322.49
25th percentile (Q_1_)	0.24	32.90	16.56
50th percentile (Q_2_)	0.86	61.99	54.23
75th percentile (Q_3_)	1.26	126.53	77.46
No. of outliers	3	2	3
Skewness	2.51	2.00	2.11

It can be noticed that all three variables are right skewed with the skewness coefficient above 2. The number of outliers can also be noticed. For identifying the outliers, the interquartile range (IQR), i.e., the difference of 75th percentile (Q_3_) and 25th percentile (Q_1_), was first calculated. The values that were higher than $Q_{3} + 1.5 \times IQR$ or lower than $Q_{1} - 1.5 \times IQR$ were considered as the outliers.

### K-means clustering

Once the normalized outage parameters were aggregated at the ZIP code level, they were used in a K-means clustering algorithm. K-means is one of the popular clustering techniques that has been widely used for clustering purposes^42–44^. K-means clustering is relatively simple compared to other clustering algorithms. Also, its computing efficiency is high. However, there are certain disadvantages associated with the K-means clustering approach. It is very sensitive to the presence of outliers in the dataset. Additionally, the user needs to specify the number of clusters for this algorithm. The 3 outage parameters are the 3 variables in the clustering analysis. As shown in Table 1, for both the count and the duration of outages, the aggregated data had 3 outliers. For the number of customers affected, there were 2 outliers present. Due to such low number of outliers, K-means clustering algorithm has been utilized in this research.

Next, the correlations between the variables were checked. The correlation coefficients between normalized outage count and normalized customers affected was 0.68. The correlation coefficient between normalized customers affected and normalized outage duration was 0.66 and it was 0.99 between normalized count and normalized outage duration. Due to extremely high correlations between normalized count and normalized outage duration, one of them was removed from the clustering analysis. Since the correlation coefficient between normalized customers affected and normalized outage duration (0.66) was lower than that of normalized count and normalized customers affected (0.68), the normalized outage count was removed from the analysis. The other two variables, i.e., normalized customers affected, and normalized outage duration were used in clustering.

Before the implementation of the clustering algorithm, the data was preprocessed. First, the variables were converted to their natural logarithm so that the skewness coefficients are between − 1 and 1. After this transformation, the skewness coefficients of normalized customers affected and normalized outage duration were − 0.30 and − 0.67, respectively. Next, the transformed variables were standardized. This step was necessary to ensure that no variable is given higher or lesser importance due to the difference in their ranges. Standardization follows Eq. (1) where $x$ = variables used in the clustering analysis, $\mu$ = mean of the variable, $\sigma$ = standard deviation of the variable, and $z$ = is the variable after standardization. It should be noted that $z$ has a mean of 0 and a standard deviation of 1. All values of $z$ range between [− 1, 1].

1 $$z = \frac{x - \mu }{\sigma }$$

In the K-means approach, each cluster is associated with a center point or centroid. Each point is assigned to the cluster with the closest centroid. The basic algorithm is simple and as follows.
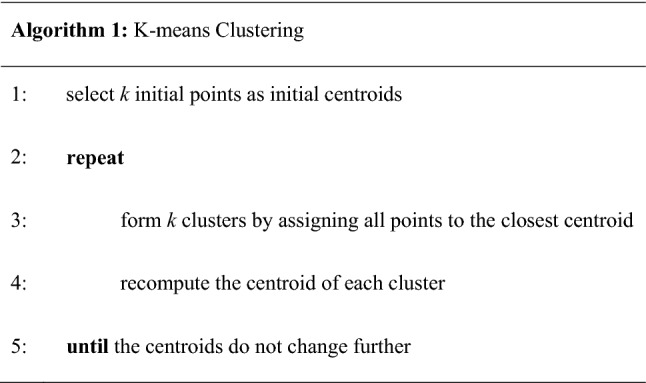


**Figure 3:**
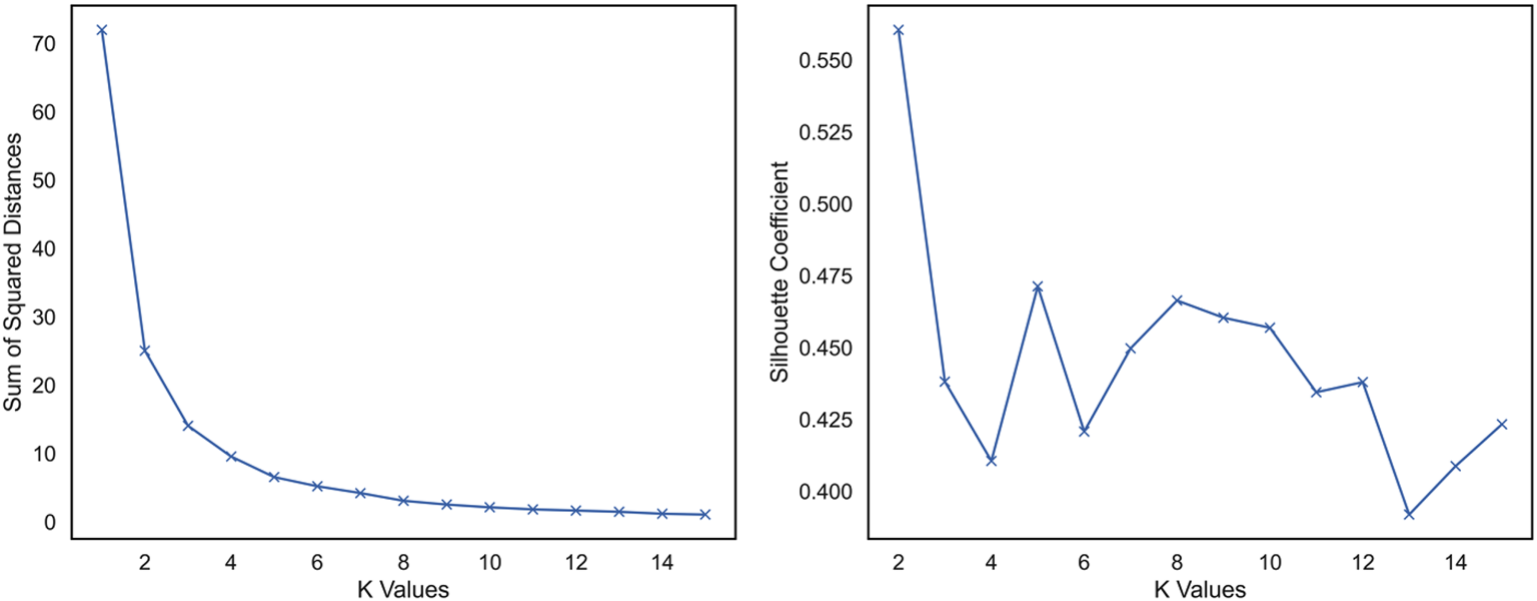
Outcomes of Elbow method.

The closeness is measured in terms of Euclidean distance, cosine similarity, correlation, etc. In this paper, Euclidean distance has been used as the measure of closeness between points. The clustering model was developed in Python programming language using the “scikit-learn” library. To determine the number of clusters, Elbow Method is widely used^44–47^. In this method, the number of clusters (*k*) is varied in a given range. For each value of *k*, the sum of squared distance (SSD) between the points in a cluster and the centroid of that cluster is measured. Then, the sum of the squared distance is plotted against *k*. The plot looks like an elbow. The point of inflection of the curve is selected as the optimal *k*. In addition to SSD, the paper has also used Silhouette Coefficient (SC) for determining the optimal *k*. The coefficient measures the goodness of the clustering technique. It is computed as Eq. (2).

2 $$Silhouette\, Coefficient = \frac{{\left( {b - a} \right)}}{{{\text{max}}\left( {a,b} \right)}}$$

where *a* = the average intra-cluster distance and *b* = the minimum of average inter-cluster distance. A Silhouette Coefficient value close to one indicates well separated clusters whereas a value close to zero indicates poor clustering performance. This paper has varied the *k* value between 1 and 15. Figure 3 shows the variation of SSD and SC for different *k*. It can be noticed that the SSD line is almost flat since *k* = 5. On the other hand, the performance of clustering algorithm measured in terms of SC is the second highest for *k* = 5 only behind *k* = 2. However, the SSD for *k* = 2 is much higher than that of *k* = 5. Considering these two factors, the number of clusters was finalized at 5.

**Figure 4:**
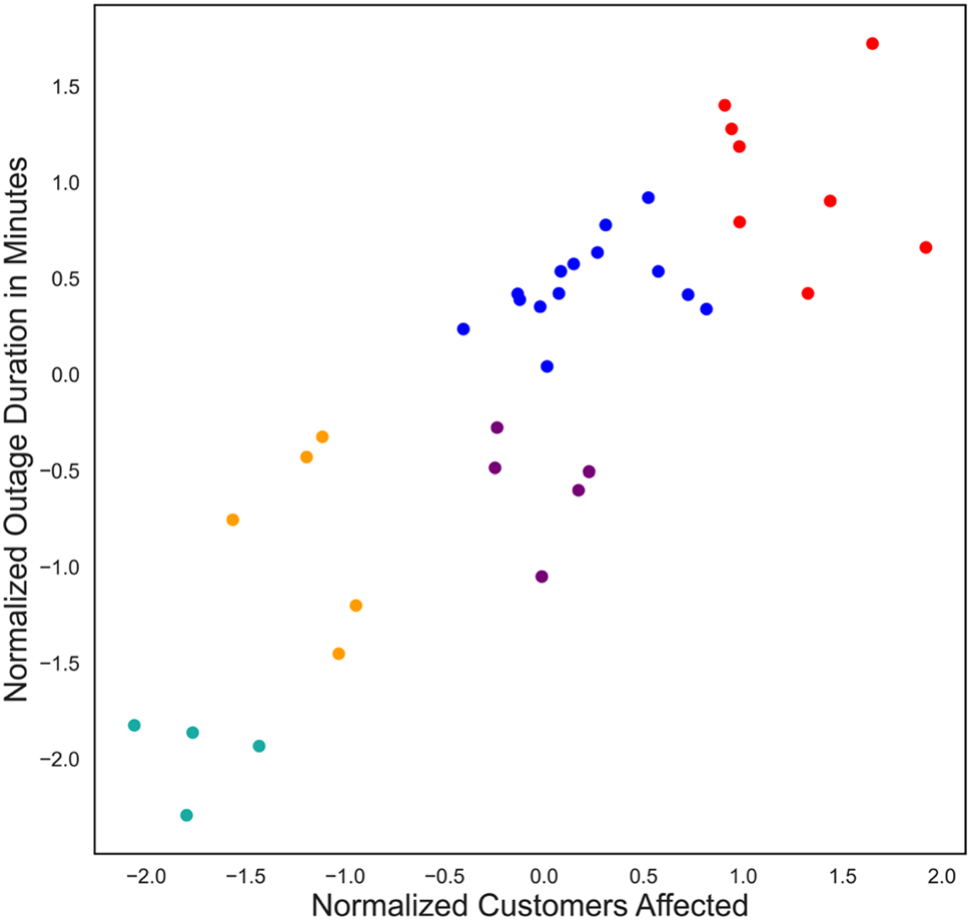
Scatter plot of the outage parameters after clustering.

Figure 4 shows the scatter plot of normalized customers affected and normalized outage duration. In the scatter plot, the five clusters of ZIP codes are shown in green, yellow, purple, blue, and red colors. It can be noticed that the values of the outage parameters have gradually increased from green cluster to red cluster. The number of ZIP codes in green, yellow, purple, blue, and red colors were 4, 5, 5, 14, and 8, respectively.

### Generalized linear models

**Figure 5:**
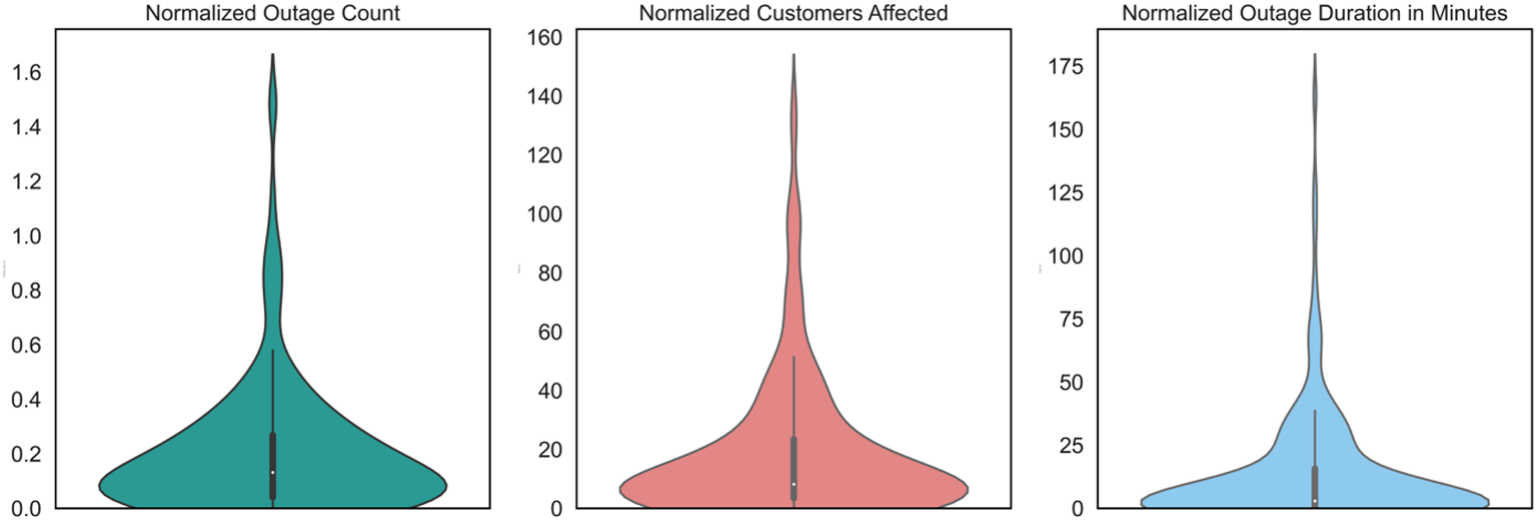
Distribution of normalized annual power outage parameters.

Since the clustering algorithm created 5 clusters of ZIP codes, the power outage data was further analyzed to see if the critical facilities, socioeconomic, and demographic characteristics of the ZIP codes can explain the outage count, number of customers affected, and duration of outages. As explained before, to control the effects of population density and location of distribution lines, the outage parameters were normalized with respect to the population density of the ZIP code. Figure 5 shows the distribution of the normalized annual outage parameters for the 36 ZIP codes for 5 years. It can be noticed that all three variables are right skewed and follows an exponential pattern. Due to this exponential nature of the normalized outage parameters, Generalized Linear Models (GLM) have been adopted to analyze the impact of the presence of critical facilities, socioeconomic, and demographic characteristics on the normalized annual outage parameters.

GLMs were popularized by McCullagh and Nelder^48^. In these models, the response variable is assumed to follow an exponential family distribution. The models were developed in Python programming language using the “statsmodel” library. There are three components of the GLM.

1. *Random component* It specifies the probability distribution of the response variable. Among the three variables, normalized count was assumed to follow a Poisson distribution. The other two variables were assumed to follow Gamma distribution.
2. *Systematic component* It specifies the linear combination of the explanatory variables.
3. *Link function* It specifies the link between the Random component and the Systematic component. For all three variables logarithmic link function has been used as it is the most used link function for both Poisson and Gamma GLMs^49^.

The mean regression structure of the Poisson and Gamma regression are shown in Eq. (3)

3 $$\log \left( \mu \right) = \alpha + \beta x$$

where normalized count ~ $Poisson \left( \mu \right)$, and normalized customers affected and normalized outage duration ~ $Gamma \left( {\mu , 1} \right)$, $\mu$ is the mean for Poisson distribution and scale parameter for Gamma distribution, 1 is the shape parameter for the Gamma distribution, $\alpha$ and $\beta$ are the regression coefficients, and $x$ is vector of the explanatory variables, i.e., critical facilities, socioeconomic, and demographic characteristics. Three types of critical facilities were considered in this analysis. They are hospitals, 911 centers, and fire stations. The data on the number of those critical facilities located at each ZIP code was collected from Department of Homeland Security’s (DHS) website. The data on the socioeconomic and demographic characteristics of the ZIP codes was collected from the U.S. Census Bureau. Two socioeconomic and two demographic variables were considered.

- *Socioeconomic* Median household income and percentage of families with income below 100% poverty level
- *Demographic* Percentage of White population and percentage of African American population

The state where the utility serves has approximately 84.2% White population, 10.2% Black or African American population, 2.7% Asian population. These three races are predominant in the 36 ZIP codes within the utility’s service territory. In 2020, the ZIP codes had an average 93.5% of White population and 3.2% of Black or African American population. In 2020, the ZIP codes had a median household income of $53,036 (lower than the national average) and an average 12.4% of the population lived in poverty (higher than the national average).

**Figure 6:**
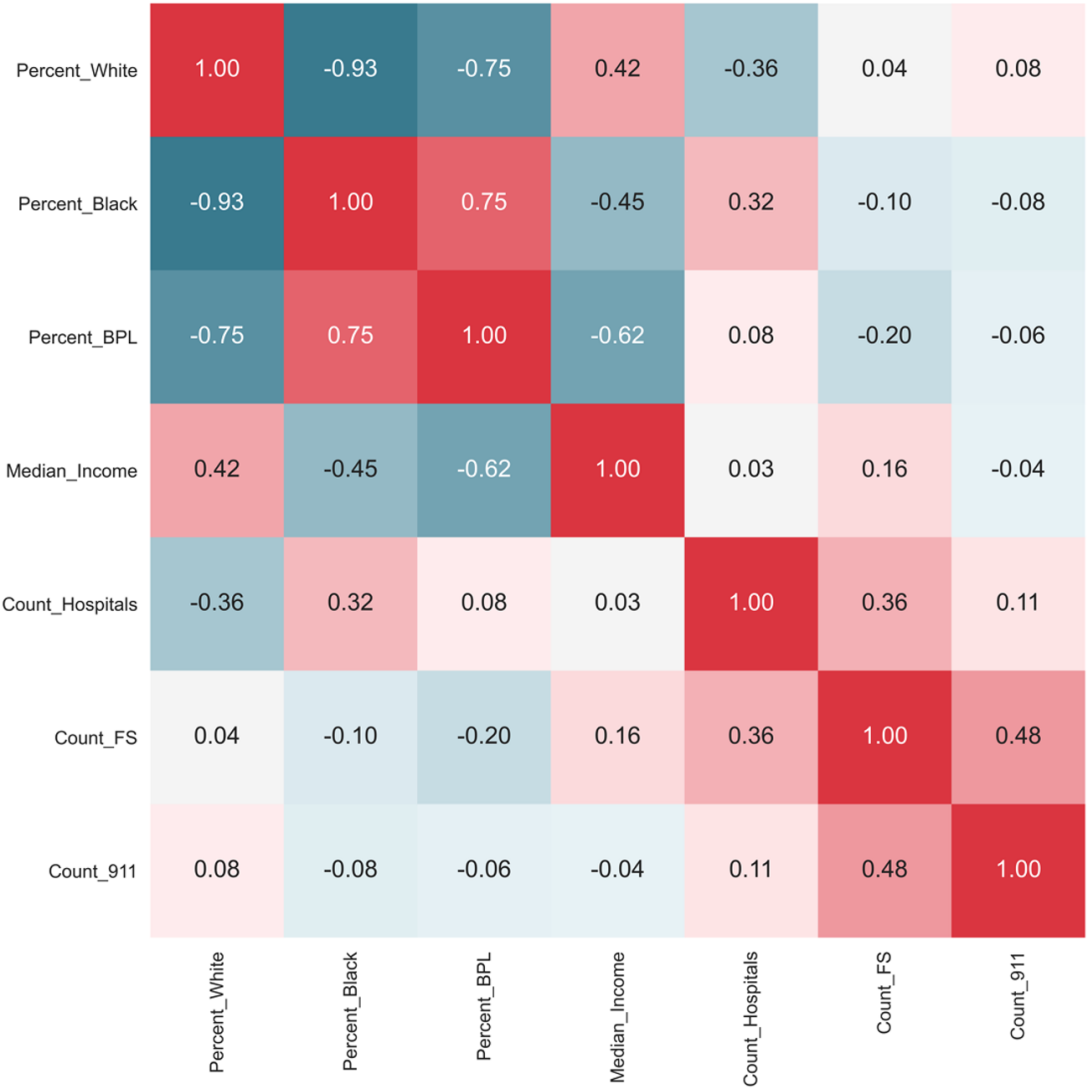
Correlation matrix for the predictors of the GLMs.

Before developing the GLMs, the correlations between the predictors were tested. Figure 6 shows the correlation matrix for the predictors. The correlation matrix shows high correlations between (1) percentage of White population and percentage of Black population, (2) percentage of White population and percentage of population living below poverty level (BPL), (3) percentage of Black population and percentage of population living below poverty level, and (4) percentage of population living below poverty level and median household income. To avoid the multicollinearity issue among the predictors, percentage of Black population and percentage of population living below poverty level were removed from the list of predictors. So, the final predictors are percentage of White population, median household income, number of hospitals, number of 911 centers, and the number of fire stations in the ZIP codes. These five predictors were used in the GLMs to test if they can explain the normalized annual outage parameters.

## Results and discussion

**Figure 7:**
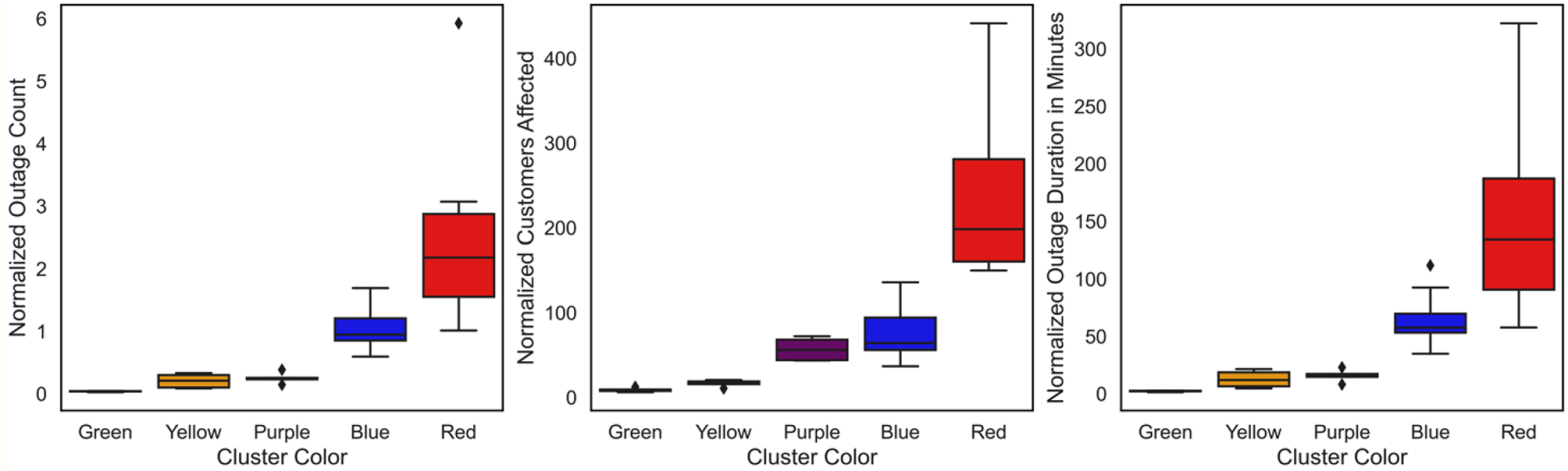
Boxplots of the normalized outage parameters aggregated over 5 years.

The clustering algorithm created 5 clusters out of the 36 ZIP codes in the utility’s service territory. The number of ZIP codes in green, yellow, purple, blue, and red clusters were 4, 5, 5, 14, and 8, respectively. Next, the statistical significance of the inter-cluster differences was tested. To do that, this research has used Kruskal–Wallis test^50^. It is a nonparametric statistical test that is used to assess the statistical significance of the difference of a single, non-normally distributed continuous variable among three or more independently sampled groups^51^. In a Kruskal–Wallis test, the null hypothesis claims that the samples are drawn from the same population, whereas the alternate hypothesis rejects that claim. The test results are shown in terms of *p*-value. If the *p*-value is less than 0.05, the test rejects the null hypothesis. For this paper, the Kruskal–Wallis test was conducted to the test the statistical significance of the difference of the 3 outage parameters among the 5 clusters. For all three parameters, the *p*-values were found in the order of 10^−6^, which is lesser than 0.05. This indicates the statistical significance of the difference. Figure 7 shows the boxplots for the 3 outage parameters for the 3 clusters.

The boxplot follows the same color convention as in Fig. 4. It can be seen that cluster shown in red color that appears on the top right corner on Fig. 4 has the highest values for all 3 outage parameters. The cluster that is shown in green and also appearing on the bottom left corner of Fig. 3 has the lowest values for all 3 outage parameters. Evidently, the 8 ZIP codes that are in the red cluster have experienced the highest extent of power outage compared to the other ZIP codes. The medians of the normalized outage parameters are shown in Table 2. It can be noticed that the medians of all 3 outage parameters have gradually decreased from clusters in red color to clusters in green color. It should be noted that the median values of the outage parameters shown in Table 2 are aggregated over five year. For instance, the 8 ZIP codes that belonged to the red cluster had a median normalized outage count, i.e., the median of the total outage count among those 8 ZIP codes over 5 years and normalized with respect to population density, of 2.18.

**Table 2:** Median of normalized outage parameters in different clusters.

Cluster color	No. of ZIP codes	Outage count	Customers affected	Outage duration in minutes
Red	8	2.18	198.58	134.28
Blue	14	0.95	64.56	57.86
Purple	5	0.24	56.28	17.06
Yellow	5	0.21	17.34	12.28
Green	4	0.04	8.50	2.71

Next, the inter-cluster difference of three popular reliability indices were compared. They are System Average Interruption Duration Index (SAIDI), System Average Interruption Frequency Index (SAIFI), and Customer Average Interruption Duration Index (CAIDI). These indices were calculated for each ZIP code on annual basis for each year between 2017 and 2021. SAIDI represents the average annual outage duration for each customers served by the utility. SAIFI is the annual number of outages per customer. CAIDI represents the average time required to restore service after an outage. Unlike SAIDI and SAIFI, CAIDI only considers the customers affected by the outage. Kruskal–Wallis test was used again to test the statistical significance of the inter-cluster difference of the reliability indices. The *p*-values for SAIDI, SAIFI, and CAIDI were 0.046, 0.000002, and 0.23, respectively. Clearly the difference in SAIDI and SAIFI were significant as the *p*-values were less than 0.05. The statistical significance of the inter-cluster difference in reliability index and the outage parameters implied differential experience with power outage in different location within the utility’s service territory.

Table 3 displays the median of reliability indices for different clusters. It can be noticed that SAIDI, SAIFI, and CAIDI are the worst, i.e., the highest in the red cluster. This means that these ZIP codes had the worst outage experiences between 2017 and 2021. The reliability indices are the best, i.e., the lowest in the five ZIP codes that belonged to yellow cluster. It can be noticed that the order of SAIDI, SAIFI, and CAIDI is different in Table 3 from that of Table 2. This is due to the difference in the area of the ZIP codes. The values in Table 2 are normalized with respect to population density, which incorporates the area of the ZIP codes. On the other hand, the values in Table 3 are normalized with respect to population only. Therefore, the difference in the area of different ZIP codes created the difference in the order in Tables 2 and 3.

**Table 3:** Median of the annual reliability indices in different clusters.

Cluster color	SAIDI	SAIFI	CAIDI
Red	28.79	0.55	70.83
Blue	8.46	0.23	41.18
Purple	6.49	0.22	30.45
Yellow	2.75	0.12	33.29
Green	8.97	0.19	64.97

In the next step, the influence of the critical facilities, socioeconomic, and demographic characteristics of the ZIP codes on the outage parameters was tested using the GLMs. Five predictors were used for developing the GLMs. They are the number of hospitals, 911 centers, and fire stations in the ZIP codes, percentage of the White population, and median household income. The GLMs were developed using the panel data. The panel data captured the annual values of the normalized outage parameters and the predictors for the 36 ZIP codes between 2017 and 2021. So, there were 36 datapoints for each year, i.e., 180 datapoints in total. As explained earlier, the normalized annual outage counts were fitted to a Poisson Regression with logarithmic link function. The other two variables, i.e., normalized annual customers affected and normalized annual outage durations in minutes were fitted to the Gamma Regression with logarithmic link function. Table 4 shows the regression coefficients of the predictors along with the *p*-values.

**Table 4:** Regression coefficients and *p*-values of GLMs.

Predictors	Normalized annual outage count		Normalized annual customers affected		Normalized outage duration in minutes	
	Coefficients	*p*-values	Coefficients	*p*-values	Coefficients	*p*-values
Intercept	− 4.33	0.15	− 0.99	0.32	− 1.29	0.32
% White population	4.58	0.15	4.44	0.00**	5.32	0.00**
Median income	− 2.18 × 10^−5^	0.08*	− 7.63 × 10^−6^	0.27	− 1.04 × 10^−5^	0.29
No. of hospitals	− 0.61	0.37	− 0.21	0.13	− 0.52	0.00**
No. of fire stations	− 0.17	0.29	0.08	0.32	− 0.18	0.08*
No. of 911 centers	0.02	0.98	0.32	0.27	0.06	0.88

* Indicates significance at 10% level.

** Indicates significance at 5% level.

**Figure 8:**
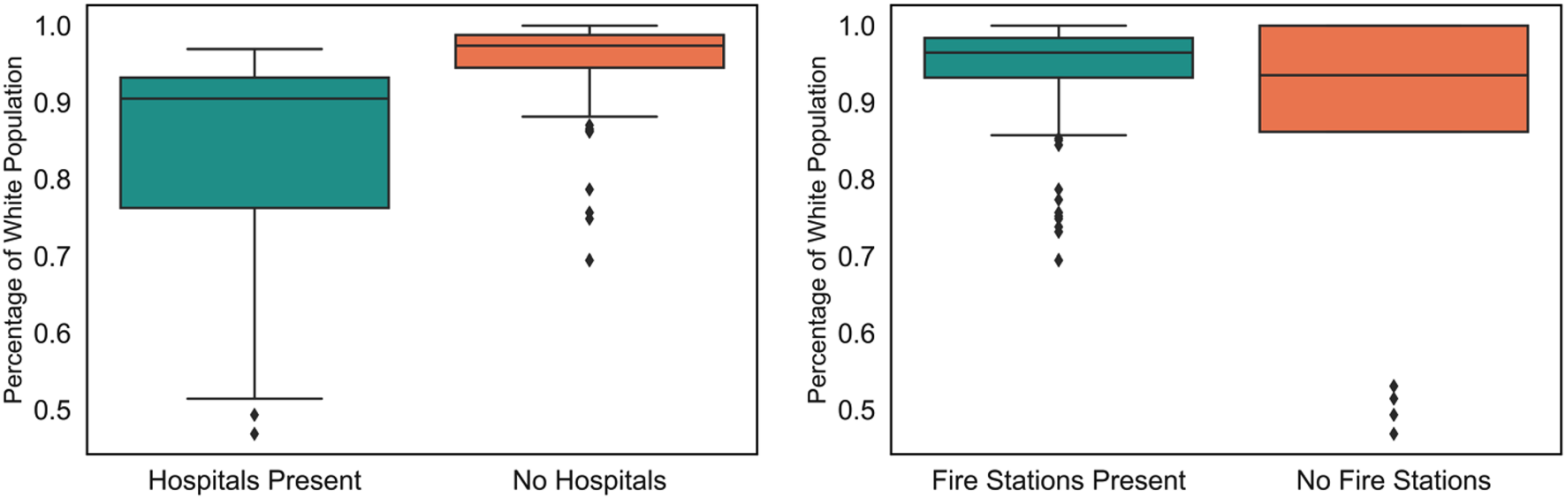
Presence of critical facilities and percentage of white population.

Table 4 shows the regression coefficients and the corresponding *p*-values. A regression coefficient has been considered significant, if the *p*-value is less than or equal to 0.10, i.e., significance level of 10%. For the normalized outage count, it can be noticed that the coefficient for median income is negative and significant. This means that the normalized annual outage count is higher in the ZIP codes with lower median household income. For the number of customers affected, the only significant predictor is the percentage of White population, and the coefficient is positive. This implies that the normalized annual customers affected is higher in the ZIP codes where the percentage of White population is higher. For the outage duration, percentage of White population, number of hospitals, and the number of fire stations are three significant explanatory variables. Out of these three, percentage of White population had positive regression coefficient, which indicates that the normalized annual outage duration is higher in the ZIP codes with higher percentage of White population. On the other hand, the coefficients of the number of hospitals and the number of fire stations are both negative. This is means that the normalized annual outage duration is higher in the ZIP codes with lower number of critical facilities. This is because restoration of critical facilities is often prioritized over the households. Figure 8 shows the variation of the percentage of White population in the ZIP codes with and without hospitals and fire stations.

It can be noticed that ZIP codes where hospitals are present have lower percentage of White population compared to the ZIP codes without any hospitals. This difference is statistically significant with *p*-value of 1.26 × 10^−11^. This result is insightful as it implies that if the utility prioritizes the ZIP codes where hospitals are present in its power outage restoration effort, it will automatically prioritize the ZIP codes with lower percentage of White population. As a result, the outage duration will be higher in the ZIP codes with higher percentage of White population. On the other hand, the variation is percentage of White population based on the presence of fire stations is statistically insignificant as the *p*-value is 0.27. Based on the results, it can be concluded that utility’s power outage restoration is unbiased. However, ZIP codes with lower median household income have experienced more power outages. Additionally, the ZIP codes with higher percentage of White population have had power outages of larger scale that have affected more customers. This can be due to spatial difference in vulnerability of grid components, which is something the utility must investigate further.

Based on the findings, this research suggests that utilities should periodically assess the performance of their outage management practice so that they can identify any bias in it. The regulatory commission should also oversee this periodic assessment neutrally. If disparities are identified, the regulatory commission should intervene immediately. These steps are necessary to ensure no community is discriminated against in the power outage management practice.

## Conclusion

This research has proposed a data driven approach to quantify the disparity in the power outage management practice of a utility. The proposed approach uses an unsupervised machine learning technique to create clusters of ZIP codes where the extent of power outage is different. The proposed approach has been illustrated for an investor-owned utility located in the Midwest region in the U.S. The power outage data was collected for 5 years between March 2017 and January 2022 for 36 ZIP codes within its service territory. The K-means clustering algorithm was adopted to create clusters of ZIP codes with similar outage parameters. Five clusters of ZIP codes were found from the clustering analysis. It was found that the difference in the extent of power outages experienced by different clusters is statistically significant. Next, three GLMs were developed to test if the differential extent of power outages can be explained by the presence of critical facilities such as hospitals, 911 centers, and fire stations, socioeconomic, and demographic characteristics of the ZIP codes. It was found that the durations of power outages are lower in the ZIP codes where hospitals and fire stations are located. However, it was found that the ZIP codes that have lower median household income have experienced more frequent power outages. Additionally, ZIP code that have higher White population have experienced more severe power outages that have affected more people. This is possibly due to difference in the vulnerability of grid components in different locations, which is something the utility must investigate to ensure equitable access of electricity to all communities. It should be noted that this research did not investigate the causes behind these disparities. It is beyond the scope of this research to judge whether the disparities are caused by intentional or unintentional biases. The proposed approach is expected to help the utilities and the regulatory commissions in identifying disparities in the existing power outage management practice so that corrective actions can be taken to ensure equitable electricity supply.

There are some limitations of the data used in this research. As explained, the durations were not available for several outages, which might have influenced the outcomes of the analysis. On the other hand, during the data cleaning, some assumptions were made. For instance, the outages that did not have location information were assumed to have happened across the utility’s service territory thus affecting all the ZIP codes. This could have influenced the research outcomes. There is one major limitation of the proposed approach. If the service territory of the utility is very large, there is a possibility that the frequency and exposure to natural hazards will be different within the service territory. In that case, it will be difficult to judge the causes of any identified disparity. They might be simply due to the difference in the frequency and exposure of the natural hazards. In such cases, county wise comparison might be more insightful. Utilities can also create groups based on similar frequency and exposure to natural hazards to neutralize their effects and then conduct clustering analysis for each group separately. This will be explored in further detail in future research.

## Data Availability

Some or all data, models, or code that support the findings of this study are available from the corresponding author upon reasonable request.
